# The effects of unilateral deprivation amblyopia on fixation stability

**DOI:** 10.3389/fnins.2026.1810727

**Published:** 2026-05-29

**Authors:** M. Nasir, X. Chen, B. M. Stanley, D. Maurer, D. I. Shore, B. Thompson, A. M. F. Wong, E. Niechwiej-Szwedo

**Affiliations:** 1 Department of Kinesiology and Health Sciences, University of Waterloo, Waterloo, ON, Canada; 2 Department of Psychology, Neuroscience and Behaviour, McMaster University, Hamilton, ON, Canada; 3 School of Optometry and Vision Science, University of Waterloo, Waterloo, ON, Canada; 4 Centre for Eye and Vision Research, Hong Kong, Hong Kong SAR, China; 5 Liggins Institute, University of Auckland, Auckland, New Zealand; 6 Department of Ophthalmology & Vision Sciences, The Hospital for Sick Children, Toronto, ON, Canada

**Keywords:** BCEA, binocular, deprivation amblyopia, microsaccade, monocular

## Abstract

**Introduction:**

Deprivation amblyopia is a neurodevelopmental disorder caused by obstruction of the visual pathway due to congenital cataracts, ptosis or corneal opacities that occur during early visual development. Visual deficits persist into adulthood even though the obstruction (e.g. cataracts) have been removed early in life. The effects of deprivation amblyopia on oculomotor control have not been studied. The present study evaluates the effects of unilateral deprivation amblyopia resulting from congenital cataracts on fixation stability.

**Method:**

Seven adults with unilateral deprivation amblyopia and 18 adults with normal vision were tested during binocular and monocular viewing. A video-based eye tracker was used to record eye position of the viewing eye(s) (closed-loop condition with visual feedback) and the covered eye (open-loop condition with no visual feedback).

**Results:**

Findings for the control group were consistent with previous studies. Fixation stability (eye position stability), evaluated using bivariate contour ellipse area (BCEA), microsaccade rate, amplitude and slow drift velocity, was best during binocular viewing, and significantly worse during open-loop monocular viewing. In comparison to the control group, patients had similar fellow eye fixation stability under binocular viewing, but fixation (eye position stability) was poorer under monocular closed-loop and open-loop viewing. Fixation stability was worst in the amblyopic eye in all viewing conditions.

**Discussion:**

Our findings demonstrate fixation stability deficits in adults with unilateral deprivation amblyopia, underscoring the lasting impact of early visual deprivation on oculomotor function.

## 1 Introduction

Deprivation amblyopia occurs when babies are born with congenital cataracts which prevent patterned light from reaching the retina (Hatt et al., 2006). Although the deprivation only lasts a short time—early surgical intervention removes the cataracts, and postoperative correction with contact lenses or glasses attempt to restore normal vision—it alters visual development and produces lifelong deficits in some visual functions. Early postnatal visual experience provides critical input to drive normal development of structural and functional connectivity in the brain (Duffy et al., 2023). When deprivation is monocular, it causes a shift in ocular dominance towards the eye providing better visual signals as the brain suppresses signals from the deprived eye (Hubel and Wiesel, 1962). This imbalance results in sensory deficits, such as decreased visual acuity and poor contrast sensitivity, as well as reduced binocular fusion and impaired depth perception (Mitchell and Maurer, 2022). While these sensory deficits are well documented, little is known about the effects of deprivation amblyopia on oculomotor control. The present study addresses this gap by assessing fixation stability in adults with monocular (unilateral) deprivation amblyopia.

Adults and children with strabismic, anisometropic or mixed amblyopia demonstrate poor fixation stability, with the greatest deficits found during monocular viewing with the amblyopic eye (Chung et al., 2015; González et al., 2012; Subramanian et al., 2013). Decorrelated binocular experience during early development due to non-deprivation amblyopia and/or strabismus is associated with involuntary, pathological eye movements such as infantile nystagmus syndrome or fusion maldevelopment nystagmus syndrome (FMNS) (Kang et al., 2019). Infantile nystagmus is characterized by involuntary, conjugate, horizontal-torsional eye movements that often accelerate in their slow phases and may transition from pendular to jerk patterns (Zahidi et al., 2017). FMNS involves conjugate, horizontal, uniplanar eye movements with linear and decelerating slow phases, often presenting as a dual-jerk waveform, where the jerk is directed toward the fixing eye (Tychsen et al., 2010). Additional pathological eye movements include involuntary saccades such as horizontal square-wave jerks, macrosaccadic oscillations, and ocular flutter (Phillipou et al., 2014). Abnormal fixational eye movements are linked to treatment outcomes (Scaramuzzi et al., 2021), therefore, it is essential to characterize fixation stability in patients with monocular deprivation amblyopia.

While attempting to hold a steady fixation, involuntary motion of the eyes consists of high frequency tremor, microsaccades, and slow drifts. These types of eye movements reduce overall fixation stability—the ability to maintain fixation on a stationary target (Krauzlis et al., 2017)—and cause dispersion from the desired fixation point, which can be quantified by the bivariate contour ellipse area method (BCEA; Castet and Crossland, 2012). A larger BCEA value represents poorer fixation stability, which can be attributed to an increase in fixational eye movements (e.g., larger microsaccade amplitude, increased rate of microsaccades or faster velocity of slow drifts) (Tarita-Nistor et al., 2009). Impaired fixation stability compromises the precision of retinal image projections, resulting in image quality degradation which may impact visual acuity. Thus, fixation stability is required for clear vision since greater motion blurs and distorts fine detail; however, small fixational eye movements are also required for accurate vision because a perfectly still image fades from perception. Optimal vision balances the need for some motion while preventing instability (Intoy and Rucci, 2020), consequently, fixational eye movements have an important functional role in supporting visual processing (Rucci and Poletti, 2015). Indeed, these eye movements are context- and task-dependent, with broad consensus that they support perceptual processing and task performance (Rucci et al., 2007; Fooken et al., 2026). Collectively, research supports that the nervous system actively regulates fixational eye movements through distributed cortical and subcortical oculomotor circuits (Tychsen et al., 2010). This control mechanism is likely impaired in individuals who experienced decorrelated binocular vision during the critical period of development. It is well established that in comparison to a matched control group, patients with amblyopia have poorer fixation stability characterized by larger BCEA and greater microsaccade amplitudes. In addition, some patients exhibit latent nystagmus, larger microsaccade amplitude and higher drift velocity during monocular compared to binocular viewing (Ghasia and Tychsen, 2024). Paradoxically, these differences are not captured by BCEA, stressing the importance of examining the microsaccade amplitude and drift velocity to gain better insights into fixation stability (Kang et al., 2019).

Examining fixation stability during binocular and monocular viewing provides insight into the role of visual feedback in fixation control. During binocular viewing, both eyes focus on a target and receive visual feedback, which is associated with better fixation stability (i.e., smaller BCEA) when compared to monocular viewing (González et al., 2012). Eye position stability of the viewing eye during monocular viewing is significantly better compared to that of the non-viewing eye because visual feedback improves fixation stability of the viewing eye through closed-loop control. In contrast, eye position stability is worse in the covered eye due to the lack of visual feedback resulting in open-loop control.

The objective of the present study was to evaluate fixation stability in patients with monocular deprivation amblyopia during binocular and monocular viewing under closed-loop (with visual feedback) and open-loop (without visual feedback) conditions. Based on a previous study using a similar experimental approach (González et al., 2012), we hypothesized that during binocular viewing the fixation stability of the patients’ fellow eye will be comparable to the control group, while the fixation stability of the amblyopic eye will be poorer due to greater positional uncertainty. During monocular viewing, we hypothesized that the fellow eye will have similar fixation stability to the control group under the closed-loop condition but poorer eye position stability in the open-loop condition due to the absence of visual feedback. We further hypothesized that the amblyopic eye will have the poorest fixation stability with an increased rate and amplitude of microsaccades and higher velocity of slow drift in all viewing conditions.

## 2 Methods

This study was approved by the University of Waterloo Office of Research Ethics and the Research Ethics Board at The Hospital for Sick Children (SickKids, Toronto, ON, Canada). Written informed consent was obtained from all participants prior to their participation.

### 2.1 Participants

Seven patients with congenital unilateral deprivation amblyopia (age 34.8 ± 8.7 years; 5 females) were recruited from a database housed at McMaster University (Hamilton, ON, Canada) and were tested at SickKids. Clinical information regarding cataract treatment was obtained from the database. Patients’ visual acuity in each eye and binocular vision were assessed on the day of the experiment. Visual acuity was assessed using a Snellen Chart. Binocular vision was assessed using the Titmus test and the Worth 4-Dot (W4D) test. The demographics and clinical information of each patient are summarized in Table 1.

**Table 1:** Summary of the demographics and clinical information of 7 patients recruited in this study.

ID	Sex	Age	Cataract diagnosis age (days)	Cataract removal age (days)	Clinical history notes	Fellow eye acuity (logMAR)	Amblyopic eye acuity (logMAR)	Stereoacuity (arcsec)	Visible strabismus (Y/N)	Worth 4 dot test
P1	F	29	160	183	Strabismus diagnosed at 6 years Received extensive patching Interocular lens inserted at 16 years	0.10	Finger count @ 30 cm	>400	Y	Suppress
P2	F	51	0	827	No history was available	0.10	Hand wave @ 30 cm	>400	N	Suppress
P3	F	29	N/A	140	Interocular lens in amblyopic eye Received extensive patching Strabismus correction procedure at 19 years	−0.10	2.0	>400	N	Suppress
P4	F	41	75	110	Several procedures for strabismus correction in both amblyopic and fellow eye Goniotomy at 8 years	−0.30	1.28	>400	Y	Suppress
P5	M	25	0	10	Strabismus correction procedure at 16 years	0.00	1.28	>400	N	Alternating suppression^a^
P6	F	31	21	42	Strabismus correction procedure at 5 years	0.10	1.63	>400	Y	Suppress
P7	M	34	2	45	Strabismus correction procedure at 1 year	0.40	1.33	>400	Y	Suppress

a During the W4D testing, the patient reported seeing 2 dots and 3 dots alternately.

The control group consisted of 18 adults (age 30.2 ± 7.25 years; 9 females) who were recruited and tested at the University of Waterloo (Waterloo, ON, Canada). Normal vision was confirmed through the Lovie-Bailey visual acuity chart (−0.15 ± 0.11 logMAR) and the Randot stereoacuity test (25.15 ± 8.12 arc seconds).

### 2.2 Equipment

Eye position was recorded using an EyeLink II eye tracker (SR Research Ltd., Mississauga, Ontario, Canada) at the University of Waterloo and an EyeLink 1000 eye tracker (SR Research Ltd., Mississauga, Ontario, Canada) at SickKids. According to the manual, the instrument noise was 0.01 deg RMS (root mean square), and both eye trackers had a spatial resolution of 0.05 deg. The eye trackers recorded at a sampling frequency of 250 Hz using the pupil and corneal reflection mode. A five-point calibration was performed under binocular viewing, followed by a validation trial to ensure error <1 deg. The fixation stimulus was presented on a Samsung LCD monitor (resolution: 1920 × 1080; refresh rate: 60 Hz; luminance of 175 cd/m^2^) at the University of Waterloo, and a BenQ LCD monitor (resolution: 1920 × 1080; refresh rate: 60 Hz; luminance of 178 cd/m^2^) at SickKids. A chinrest and a headrest were used to stabilize the position of the participants’ head.

### 2.3 Procedure

Participants were seated 60 cm from the monitor. The test was conducted in a well illuminated room. Each trial began with a centrally presented 3 deg black bullseye and crosshair fixation stimulus presented on a white background for 20 s. The fixation stimulus was selected because a previous study recommended it as the optimal stimulus for fixation stability protocols (Thaler et al., 2013). Participants were instructed to fixate on the center of the fixation target. The inter-trial interval was at least 20 s.

Participants were tested under three viewing conditions: binocular, monocular dominant (fellow) eye viewing, and monocular non-dominant (amblyopic) eye viewing. In the monocular conditions, an infrared long-pass filter (cut-on wavelength ≈ 780 nm) was used to block visual input to the covered eye while still allowing the eye tracker to record eye position. Hence, in each monocular condition, the viewing eye was recorded with visual feedback (i.e., closed-loop condition), while the covered eye was recorded without visual feedback (i.e., open-loop condition). The protocol consisted of three trials under each viewing condition which were randomized across participants.

### 2.4 Analysis

All trials were visually inspected to ensure signal quality. When a blink was detected, 250 ms of the signal before and after the blink was removed to eliminate blink-associated signal artefacts (Chung et al., 2015; González et al., 2012). In addition, the first 5 s of each fixation period were removed from the analysis. Therefore, fixation stability was evaluated based on a 15-s recording in each trial.

Fixation stability was first quantified by calculating the BCEA metric, which represents the area in which the eyes are found during 68% of the recording period (Castet and Crossland, 2012). It is calculated using the following formula:


$$\text{BCEA}=2\chi^{2}\pi \sigma_{H}\sigma_{V}\sqrt{1-\rho^{2}}$$

where $\sigma_{H}$ and $\sigma_{V}$ represent the standard deviation (SD) of eye positions along the horizontal and vertical axes, respectively; *ρ* represents the product–moment coefficient, and *χ*^2^ = 2.291 is the chi-square value corresponding to the probability value of 0.68 (Castet and Crossland, 2012). BCEA data were log (base10) transformed to meet normality assumptions as in previous papers (González et al., 2012). Next, the rate and amplitude of microsaccades were analyzed using the following criteria: velocity greater than 20 deg/s and an amplitude greater than 0.1 deg (González et al., 2012). Because microsaccades are conjugate in adults with normal vision (Otero-Millan et al., 2014), only microsaccades detected in both the left and right eye recordings were analyzed for the control group. Microsaccades that vary in amplitude between the two eyes are more likely to occur in the patient group due to the acuity differences between the fellow and amblyopic eyes. Therefore, for the patient data, each microsaccade was visually inspected to confirm it was a valid microsaccade. Intrusive saccades (> 2 deg in magnitude) were not included in the microsaccade analysis. Ocular slow drift was quantified by the mean eye velocity during the fixation interval between microsaccades. Slow drift duration of <100 ms was removed from the analysis because short durations do not provide a reliable estimate of the velocity.

### 2.5 Statistical analysis

First, the control group data were analyzed to establish the typical range for fixation stability across viewing conditions and the effect of visual feedback (i.e., closed- vs. open-loop). Normality of the data was confirmed using the Shapiro–Wilk test, homogeneity of variance was confirmed using Levine’s test, and sphericity was confirmed using Mauchly’s test of sphericity. Each measure of fixation stability (BCEA, microsaccade amplitude, and mean slow drift velocity) was subjected to a two-way repeated measures analysis of variance (ANOVA) with three viewing conditions (binocular, monocular closed-loop, monocular open-loop) and eye (dominant, non-dominant). Only conjugate saccades were included in the analysis, therefore, microsaccade rate was the same for the dominant and non-dominant eyes. Accordingly, a one-way repeated measures ANOVA was conducted to assess microsaccade rate across the three viewing conditions (binocular, monocular closed loop, monocular open loop). The control group’s mean and standard deviation (SD) for BCEA, microsaccade rate, microsaccade amplitude, and slow drift velocity were subsequently used to calculate *Z*-scores used to compare against the patient group. Given the heterogeneity and our relatively small sample size, this approach allowed us to determine the magnitude of the difference for each patient. Any patient *Z*-scores greater than two indicate scores outside the 95% confidence interval of the control mean.

## 3 Results

**Figure 1:**
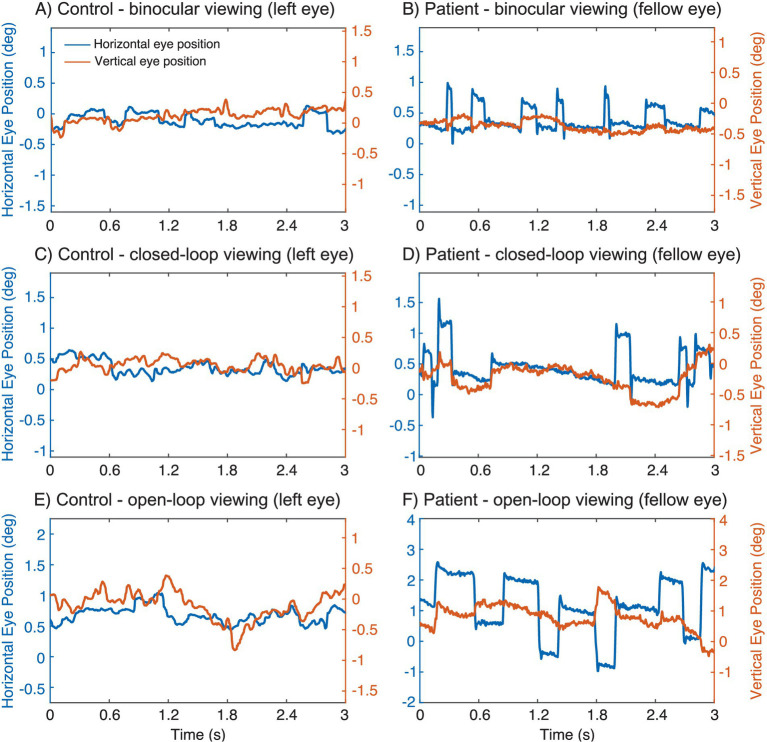
Exemplary trials showing eye position trajectories for one control participant and fellow eye position for one patient (P4) under different viewing conditions. Blue curves represent horizontal eye position; orange curves represent vertical eye position. Eye position during binocular viewing for the control participant **(A)** and fellow eye of the patient **(B)**. Eye position during monocular viewing closed-loop for the control participant **(C)** and the patient **(D)**. Eye position during monocular viewing open-loop for the control participant **(E)** and the patient **(F)**. Gaps in eye position trajectory were due to blinks. Note that the *y* range in panel **F** is doubled (6 deg) due to larger eye movements. All other panels are shown with a *y* range of 3 deg. For more illustrations, please refer to the Supplementary material.

For the control group, 78% of trials were included in the analysis and 22% were removed from analysis due to loss of tracking (i.e., blinks or artefacts). Due to difficulties with eye tracking in the patient group, 57% of all attempted trials were included in the analysis (See Supplementary Table 1 for details regarding the number of trials included in the analysis across viewing conditions). Figure 1 depicts eye position trajectories during 3 s of the fixation period for one control participant and one patient (P4) during binocular and fellow eye viewing during the closed and open-loop conditions. The fellow eye fixation stability during binocular and closed loop condition was affected due to a higher rate of microsaccades. Intrusive saccades with amplitudes >2 deg were evident during the open-loop condition. Intrusive saccades were also evident during the amblyopic eye viewing condition, which are shown in the Supplementary material, along with example trials for other patients across viewing conditions.

### 3.1 Control group

**Figure 2:**
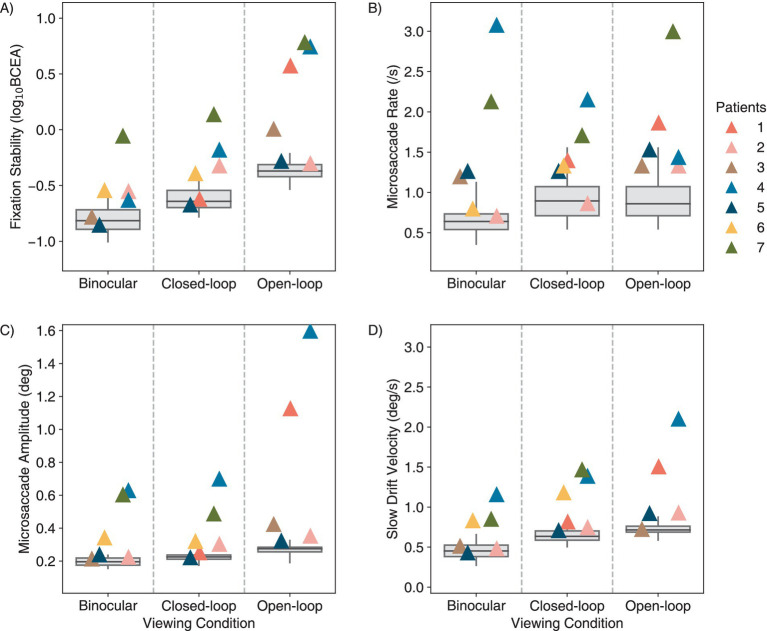
Fixation stability results for control group and the fellow eye of patients, plotted for each type of viewing condition and visual feedback availability. **(A)** Fixation stability (illustrated by log_10_BCEA). **(B)** Microsaccade rate. **(C)** Microsaccade amplitude. **(D)** Slow drift velocity. Results for the control participants are depicted by boxplots. Boxplot represents the distribution of data in the control group under each viewing condition. The horizontal line inside the boxplot represents the median. The box represents the interquartile range (25th–75th percentile), while the whiskers extend to the 5th and 95th percentiles. For control group monocular viewing, results for the left and right eyes were collapsed as there were no differences between the eyes. Individual patient’s fellow eye results are shown by different colors. In panels **C,D**, P7 data points for the open-loop condition were not included because the results were >40 standard deviations away from the control means.

Summary results for the control participants are presented using boxplots in Figure 2. Panel A illustrates the BCEA results. The repeated measures ANOVA revealed a significant main effect of viewing condition for log_10_BCEA [*F*(2,34) = 275.77, *p* < 0.001, partial *η*^2^ = 0.94], and no significant eye effect or interaction. Bonferroni corrected pairwise post-hoc comparisons revealed that fixation stability was significantly better during binocular viewing (log_10_BCEA: mean −0.814 ± SD 0.122 deg^2^) compared to closed-loop monocular viewing (−0.620 ± 0.112 deg^2^, *p* < 0.001), and open-loop condition (−0.372 ± 0.092 deg^2^, *p* < 0.001).

Microsaccade rate and amplitude results are illustrated in Figures 2B,C, respectively. Results demonstrated a significant main effect of viewing condition on microsaccade rate [*F*(2,34) = 5.40, *p* = 0.007, partial *η*^2^ = 0.24], and no significant eye effect or interaction. *Post-hoc* comparisons showed a significantly lower microsaccade rate during binocular viewing (0.68 ± 0.26 microsaccades/s) compared to monocular closed-loop and open-loop viewing (0.95 ± 0.29 microsaccades/s; 0.94 ± 0.30 microsaccades/s, respectively, *p* < 0.001). A significant main effect of viewing condition was found for the microsaccade amplitude [*F*(2,34) = 46.52, *p* < 0.001, partial *η*^2^ = 0.73], and no significant eye effect or interaction. Bonferroni corrected pairwise *post-hoc* comparisons indicated that microsaccade amplitude was not significantly different between binocular and closed-loop viewing (0.196 ± 0.027 deg; 0.229 ± 0.036 deg, respectively, *p* > 0.05), but was significantly higher during open-loop viewing (0.268 ± 0.037 deg, *p* < 0.001).

Slow drift velocity is shown in Figure 2D. Repeated measures ANOVA revealed a significant main effect of viewing condition [*F* (2,34) = 48.75, *p* < 0.001, partial *η*^2^ = 0.74], and no significant eye effect or interaction. Bonferroni corrected pairwise post-hoc comparisons indicated that slow drift velocity was significantly greater during closed-loop monocular (0.64 ± 0.09 deg/s) and open-loop monocular (0.73 ± 0.09 deg/s) compared to binocular viewing (0.46 ± 0.12 deg/s, *p* < 0.05 for both comparisons). However, the two monocular conditions did not show significant differences.

### 3.2 Patient group

**Figure 3:**
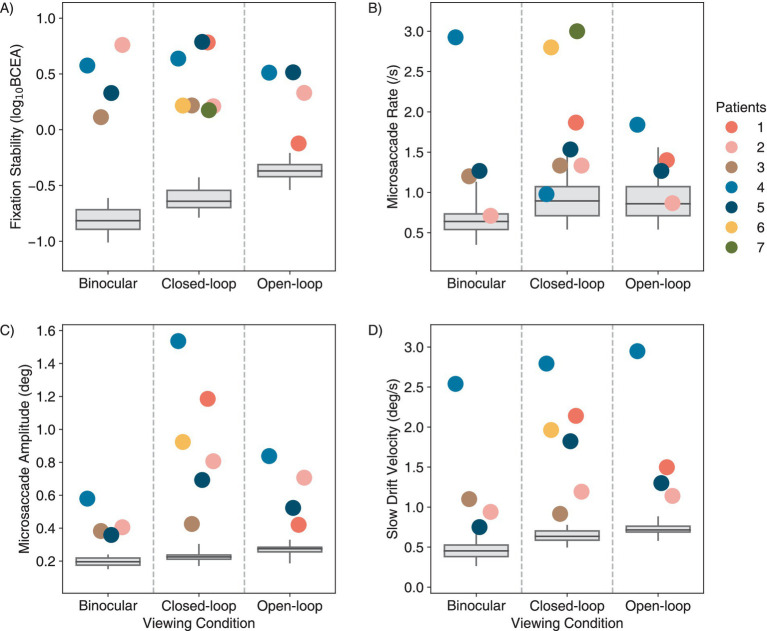
Fixation stability results for controls and the amblyopic eye of patients, plotted for each type of viewing condition and visual feedback availability. **(A)** Fixation stability (illustrated by log_10_BCEA). **(B)** Microsaccade rate. **(C)** Microsaccade amplitude. **(D)** Slow drift velocity. Results for the control participants are depicted by the boxplots for each viewing condition and are the same as those shown in Figure 2. Individual patients’ amblyopic eye results are shown by different colors. In panels **C,D**, P7 data points for the closed-loop conditions were not included because the results were > 40 standard deviations away from the control means.

Results for individual patients are plotted in Figure 2 (fellow eye) and Figure 3 (amblyopic eye) to illustrate their performance with respect to the control group. Table 2 presents a summary of the median *Z*-scores for the two eyes across viewing conditions for the patient group.

**Table 2:** Median *Z*-scores for fixation stability measures for the patient group.

Viewing condition	Eye	BCEA	Microsaccade amplitude	Microsaccade rate	Slow drift velocity
Binocular	Fellow	1.81 (1.60)	**3.56** (11.53)	**2.11** (3.84)	1.74 (2.90)
	Amblyopic	**10.22** (2.80)	**7.34** (2.68)	**2.11** (2.28)	**4.54** (4.62)
Monocular closed-loop	Fellow	**2.33** (3.05)	**2.33** (5.01)	1.41 (1.17)	**3.87** (6.09)
	Amblyopic	**7.36** (4.37)	**19.21** (16.90)	1.97 (3.37)	**14.09** (10.21)
Monocular open-loop	Fellow	**7.10** (9.73)	**13.49** (29.43)	1.80 (1.39)	**5.62** (11.70)
	Amblyopic	**8.48** (3.16)	**9.19** (6.42)	1.30 (1.12)	**7.64** (6.83)

Numbers in bold indicate large deviations from the control group (*Z*-score > 2). Interquartile ranges (IQR) are noted in brackets.

#### 3.2.1 Fellow eye fixation stability

Under binocular viewing, fellow eye BCEA and slow drift velocity were within 2 standard deviations of the control mean. However, median *Z*-scores for microsaccade amplitude and rate were greater than 2, indicating that fellow eye fixation stability was poorer in comparison to the control group. Under monocular closed-loop viewing (i.e., with visual feedback), fellow eye exhibited poorer fixation stability with a median BCEA *Z*-score of 2.33. This reduction in fixation stability was due to increased microsaccade amplitude and increased drift velocity. Under monocular open-loop viewing (i.e., without visual feedback), the fellow eye exhibited very poor eye position stability, with the BCEA falling more than 7 standard deviations away from the control group. Higher microsaccade amplitude and elevated slow drift velocity contributed to the increase in overall eye position dispersion.

*Amblyopic Eye Fixation Stability*: During binocular viewing, the amblyopic eye exhibited poor fixation stability with a median BCEA *Z*-score of 10.22, with high microsaccade amplitude, rate, and drift velocity. In the closed-loop condition, the amblyopic eye also had poor fixation stability as demonstrated by a median BCEA *Z-*score of 7.36, which was primarily attributed to high microsaccade amplitude and drift velocity. Eye position stability of the amblyopic eye during the open-loop monocular condition (i.e., fellow eye viewing) had a median *Z*-score of 8.48, also due to high microsaccade amplitude and drift velocity.

## 4 Discussion

This study sought to characterize the effects of unilateral deprivation amblyopia on fixation stability in adults. Patients tested in the current study had a history of unilateral congenital cataracts, which were removed within 2 years after birth to restore vision. As a result, all patients had very poor visual acuity in the affected eye; however, all of them were able to discern the fixation stimulus. Moreover, six of the seven patients showed suppression on the Worth 4-Dot test indicating no binocular interactions. The fixation results are mostly consistent with our hypotheses and demonstrate that fixation stability of the fellow eye is dependent on viewing condition, while the amblyopic eye exhibited significantly poorer fixation stability across all viewing conditions.

Consistent with previous research with visually normal adults (González et al., 2012), binocular viewing was associated with the best fixation stability as evidenced by fewer microsaccades, small microsaccade amplitude and low velocity slow drift. Monocular viewing impaired fixation stability but visual feedback played a critical role as demonstrated by significantly poorer eye position stability during the open-loop compared to closed-loop condition. Overall, these results confirm the significance of visual feedback in maintaining fixation stability in individuals with normal vision.

In accordance with our hypothesis and previous studies that examined strabismic and anisometropic amblyopia (González et al., 2012; Chung et al., 2015), fixation stability in patients with deprivation amblyopia was poorer overall, although the impairment varied across viewing conditions. In patients with amblyopia, the eye with better visual acuity is referred to as the fellow eye. It is important to note that even when the visual acuity of the fellow eye is normal, deficits in visual functions have been demonstrated, for example, reduced contrast sensitivity at high spatial frequencies, higher thresholds for global motion, and reading deficits (Meier and Giaschi, 2017; Birch et al., 2019; Giaschi et al., 2024). In our study, all patients (except P7) had normal VA (≤0.1 logMAR) in the fellow eye. Fixation stability of the fellow eye under binocular viewing was similar to the control group except for P7. For the other patients, fixation stability measured by BCEA was within or near 2 standard deviations of the control group, even though their microsaccade rate and slow drift velocity were slightly elevated. Notably, fellow eye fixation stability deteriorated during monocular closed-loop condition (i.e., despite fellow eye viewing), indicating that the lack of binocular visual input impacted the fellow eye fixation stability mechanism. Similar results were reported by González et al. (2012) in a cohort of adults with strabismic and anisometropic amblyopia. It should, however, be noted that most of the patients in González et al. (2012) had residual stereopsis and fusion whereas our cohort had severely compromised binocular vision. The finding that fellow eye fixation stability was better during binocular compared to closed-loop monocular condition (fellow eye viewing) suggests the possibility of residual interactions between the eyes even in cases when one eye appears to be suppressed on clinical tests. This interaction between the two eyes can be influenced by retinal (i.e., visual) and extraretinal (i.e., afference and motor efference) inputs (Ackerley and Barnes, 2011), and the role of these factors should be examined in future studies.

Consistent with our hypothesis, patients had poor position stability in the fellow eye during the open-loop condition in comparison to the closed-loop condition. Interestingly, recording of the fellow eye in the open-loop condition (i.e., amblyopic eye viewing) revealed a similar pattern of eye position instability (e.g., square wave jerk or saccadic oscillations; please see the Supplementary material) to that seen in the amblyopic eye during the same viewing condition (i.e., amblyopic eye closed-loop viewing). Thus, the poor stability of the fellow eye under open-loop condition seems to be directly associated with the loss of fixation control in the amblyopic eye. Specifically, when viewing with the amblyopic eye, retinal feedback is compromised resulting in poor stability control. Thus, in the absence of retinal input to the fellow eye, the motor efference driving the amblyopic eye’s position adversely influences the stability of the fellow eye under the open-loop recording.

The poor fixation stability in the amblyopic eye during binocular and closed-loop monocular viewing conditions may be attributed to very poor visual acuity of that eye. As shown in a previous study, poor visual acuity correlates with poor fixation stability (Chung et al., 2015). It has been proposed that there is increased random internal noise within the amblyopic visual system where neural firing patterns in response to visual stimuli are more variable, which ultimately results in increased positional uncertainty, thus limiting the spatial resolution of the visual system (Levi et al., 2007, 2008). The poor fixation stability of the amblyopic eye during binocular viewing and the closed-loop condition suggests that the system is not tuned to using visual input for oculomotor control. An intriguing possibility is that the global deficits in fixation stability could be attributed to a loss of a reference point for the oculomotor system, a hypothesis that has been proposed to explain the loss of fixation control due to macular diseases (Sverdlichenko et al., 2022). Deficits in spatial vision and poor fixation stability are the two hallmarks of macular degeneration and amblyopia, thus, the underlying mechanism might be similar in which the visual impairment causes a loss of a reference point for the oculomotor system (Tarita-Nistor et al., 2009). In case of macular degeneration, the loss of a reference point is at the level of the retina, whereas in the case of amblyopia it is a cortical problem.

In contrast to the fellow eye, microsaccade amplitude and drift velocity for the amblyopic eye under open-loop (fellow eye viewing) was significantly better compared to the closed-loop condition (amblyopic eye viewing). In this case, retinal and extraretinal inputs from the fellow eye most likely contributed to a significant improvement in the amblyopic eye position stability during the open-loop condition. Overall, this pattern of results suggests that there are interactions between the two eyes that contribute to fixation stability control.

This is the first study to demonstrate the effect of unilateral deprivation amblyopia on oculomotor control. However, there are some limitations including a small sample size, heterogeneity within the patient population, and technical challenges with eye tracking. The small sample size of the patient group (*n* = 7) is the most significant limitation in the current study. Recruitment of patients was difficult due to the rarity of unilateral deprivation amblyopia and the attrition rate in our database of patients willing to participate in studies as they age. While the sample size was smaller than ideal, the results provide an initial signal suggesting a significant impact of unilateral deprivation amblyopia on oculomotor control. Although all patients had congenital unilateral deprivation amblyopia and poor visual acuity in the amblyopic eye, individual patients presented with different oculomotor problems such as square wave jerks, dissociated nystagmus, or strabismus. This underscores the importance of future studies to test a larger cohort to validate the findings of this study. Lastly, calibrating the eye tracker in patients with unilateral deprivation amblyopia was challenging in some viewing conditions. For example, two patients presented with manifest strabismus during the binocular viewing condition, which made it difficult to track the deviated eye during binocular viewing (i.e., it was outside the range of the eye tracker recording range). Addressing these limitations in future research is crucial for advancing our understanding of oculomotor control in patients with unilateral deprivation amblyopia.

Understanding oculomotor abnormalities in deprivation amblyopia is an important step towards developing targeted interventions to improve visual function and quality of life in affected individuals. The observed patterns of poor fixation stability, abnormal microsaccade characteristics, and variability in slow drift velocity highlight the complex interplay between sensory and motor aspects of amblyopia. Future research exploring the underlying mechanisms driving this oculomotor behavior may lead to novel therapeutic approaches aimed at optimizing visual and motor outcomes in patients with amblyopia.

## Data Availability

The raw data supporting the conclusions of this article will be made available by the authors, without undue reservation.
