# Supplementary material for: The effects of unilateral deprivation amblyopia on fixation stability
Source: Front Neurosci. 2026 May 29;20:1810727. doi: 10.3389/fnins.2026.1810727 (PMC13260333; doi:10.3389/fnins.2026.1810727)
Supplement: Supplementary file 1 [file Supplementary_file_1.pdf]

The Effects of Deprivation Amblyopia on Fixation Stability and Optokinetic Nystagmus

Supplementary Table 1. Summary of the number of trials for fixation stability that were included for each patient across viewing conditions (maximum number of trials per condition was 3).

Participant	Binocular Fellow	Binocular Amblyopic	Closed-loop Fellow	Closed-loop Amblyopic	Open-loop Fellow	Open-loop Amblyopic
P1	0	0	2	2	2	2
P2	2	2	2	2	1	1
P3	3	3	0	2	2	0
P4	3	3	3	3	3	3
P5	3	3	2	1	1	2
P6	2	0	2	2	0	0
P7	2	0	2	2	2	0
