# Supplementary material for: The effects of unilateral deprivation amblyopia on fixation stability
Source: Front Neurosci. 2026 May 29;20:1810727. doi: 10.3389/fnins.2026.1810727 (PMC13260333; doi:10.3389/fnins.2026.1810727)

The Effects of Unilateral Deprivation Amblyopia on Fixation Stability

Nasir, M., M.Sc., Chen, X., Ph.D., Stanley, B. M., Ph.D., Maurer, D., Ph.D., Shore, D. I., Ph.D., Thompson, B., Ph.D., Wong, A., M.D., Ph.D., Niechwiej-Szwedo, E., Ph.D.

Supplementary Document: Eye position trajectories for individual patients.

One representative trial was selected for each viewing condition for each patient. In each graph, blue traces show horizontal eye position, and orange traces show vertical eye position. Key features are labelled for each graph. To facilitate comparison across patients and conditions, all figures use a uniform *y* range of 6 degrees, with the exception of P7 amblyopic eye viewing, which uses a doubled *y* range (12 degrees).

P1

Binocular viewing: vertical nystagmus

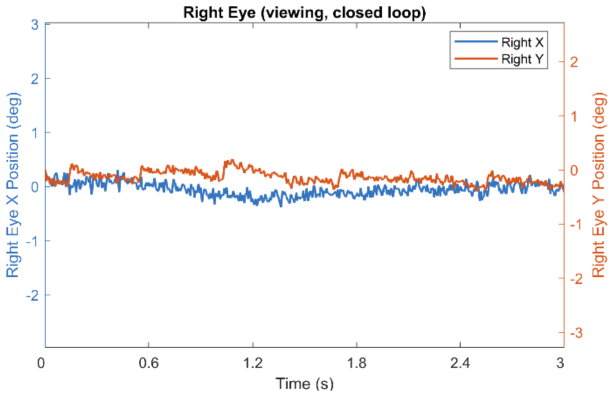

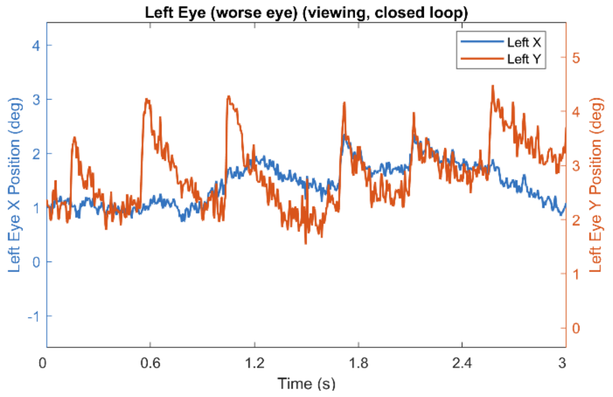

Fellow eye viewing: vertical nystagmus

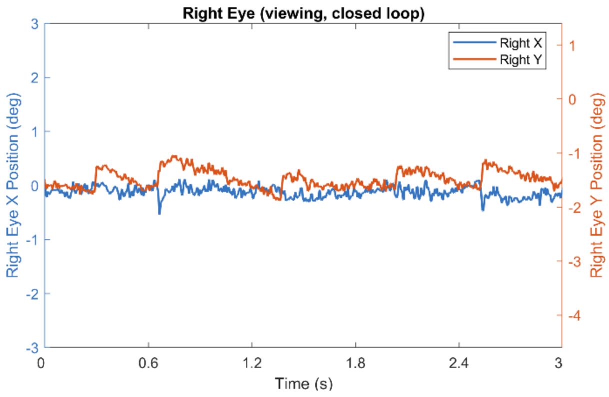

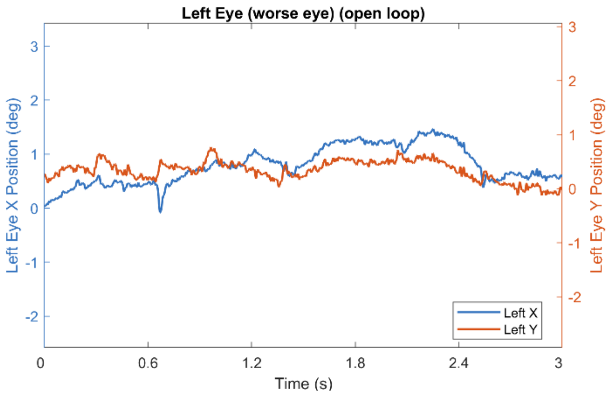

Amblyopic eye viewing: microsaccades

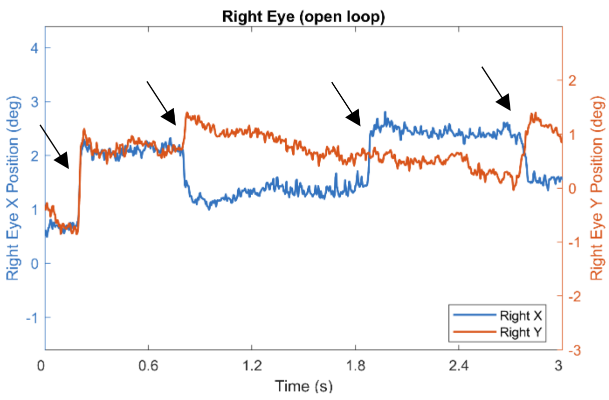

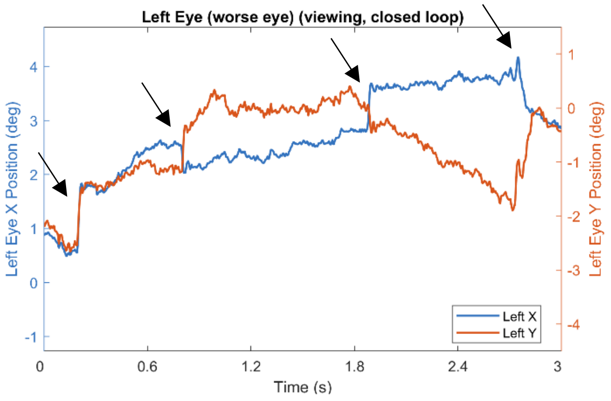

P2

Binocular viewing: horizontal nystagmus

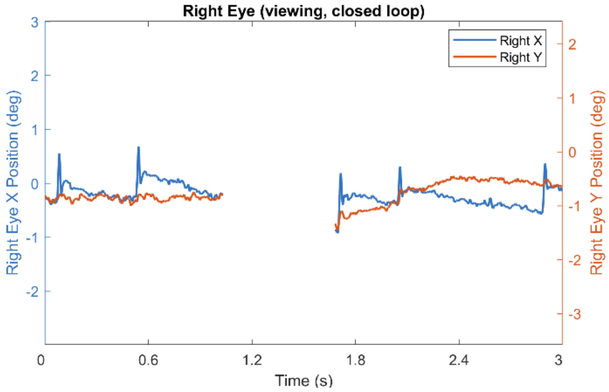

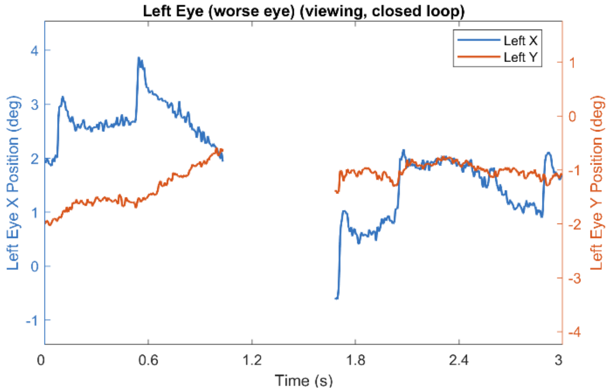

Fellow eye viewing: microsaccades (black) + horizontal nystagmus (green)

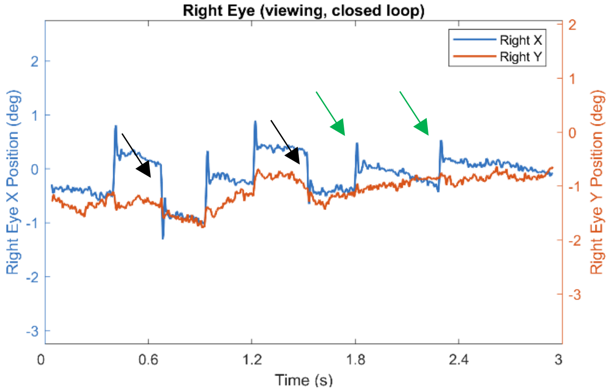

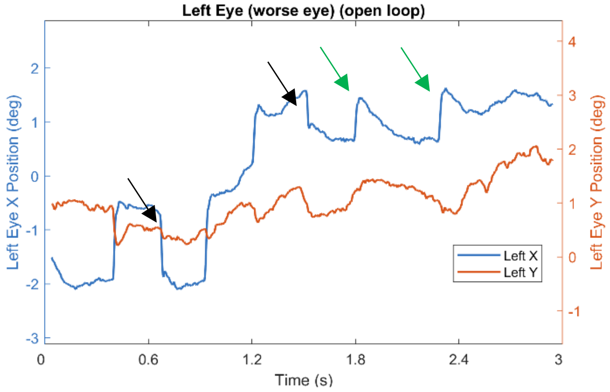

Amblyopic eye viewing:

No available data.

P3

Binocular viewing: microsaccades

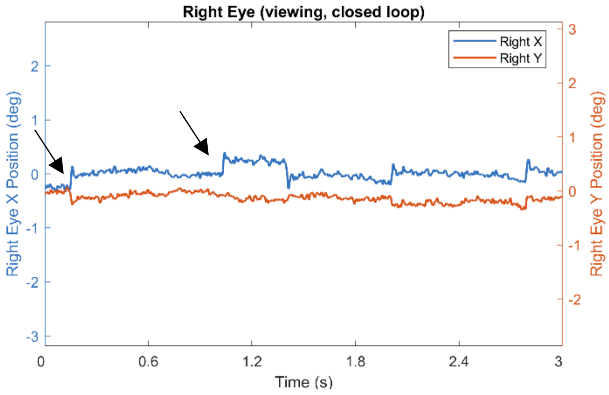

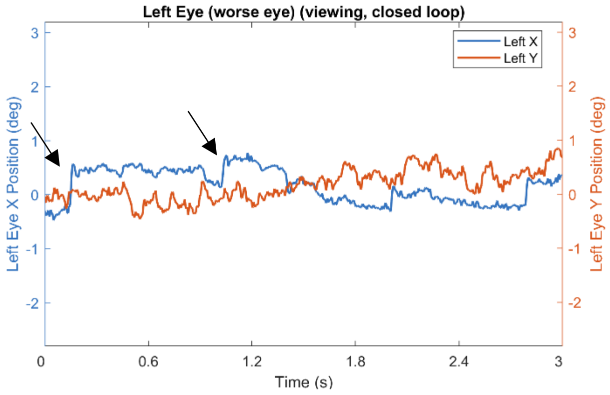

Fellow eye viewing: microsaccades

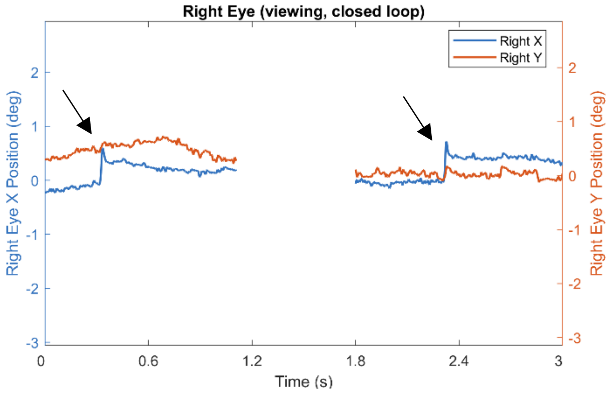

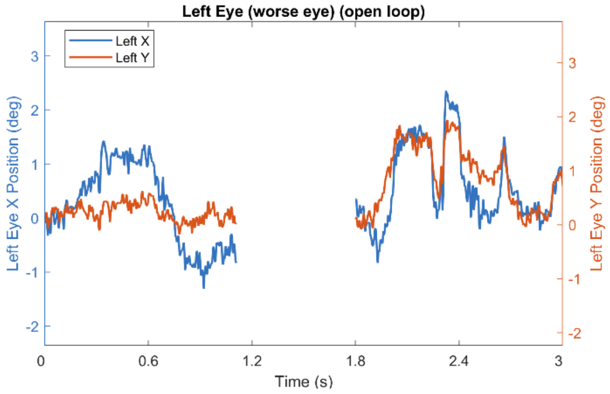

Amblyopic eye viewing: microsaccades

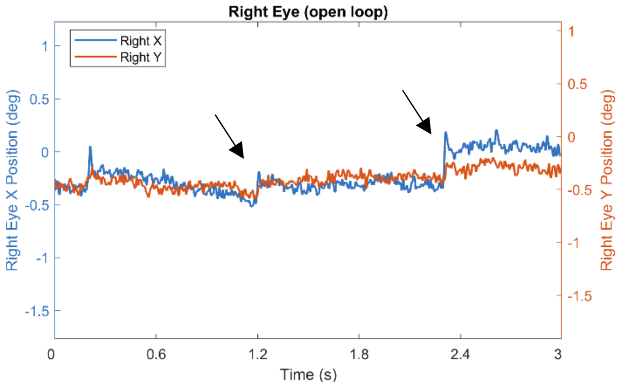

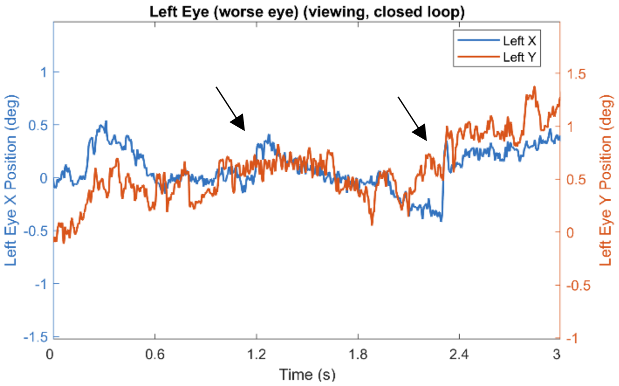

P4

Binocular viewing: square wave jerks

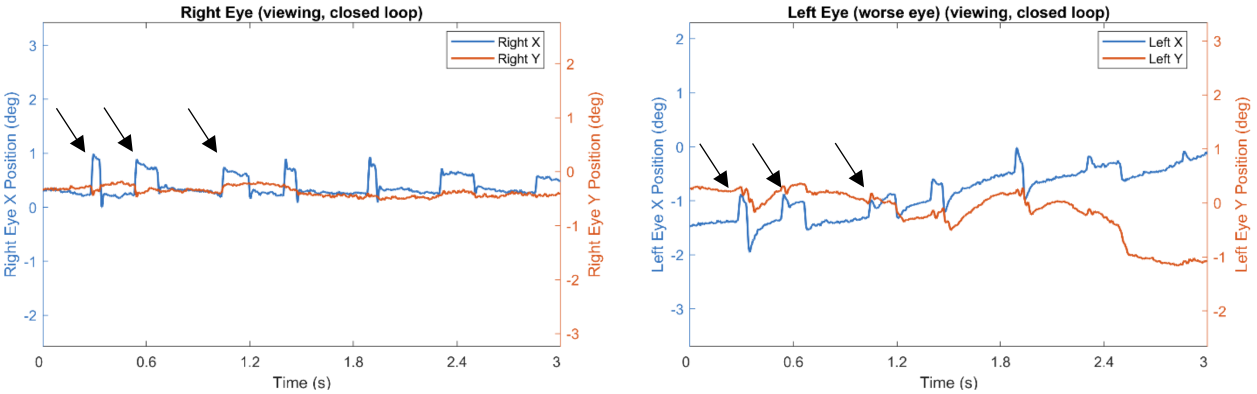

Fellow eye viewing: square wave jerks

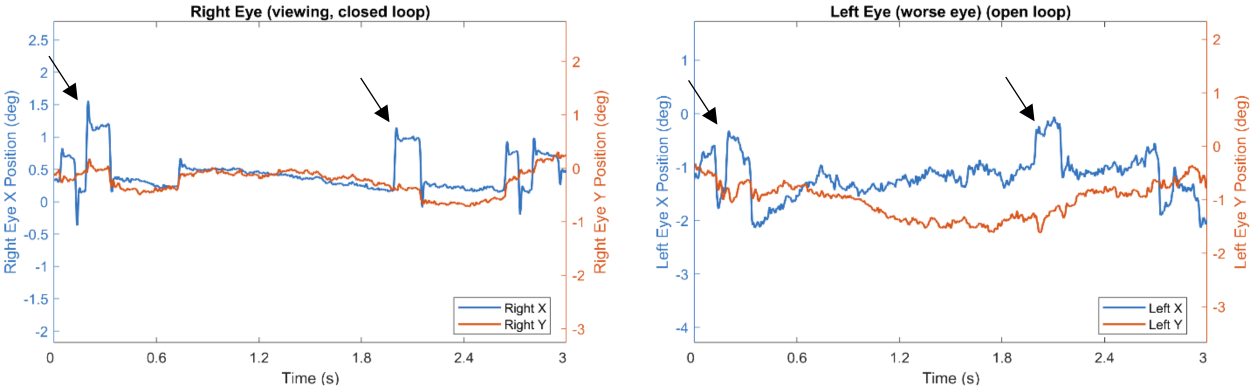

Amblyopic eye viewing: macrosaccadic oscillations

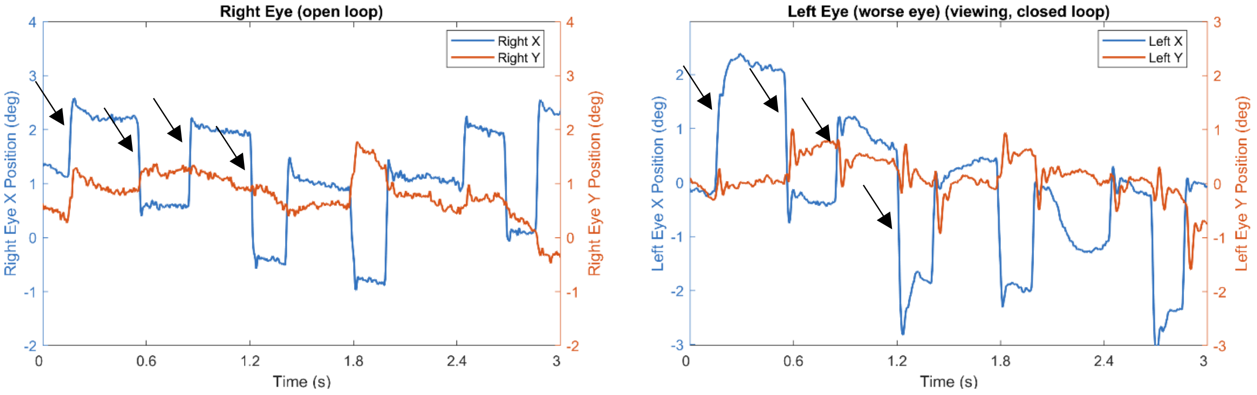

P5

Binocular viewing: nystagmus

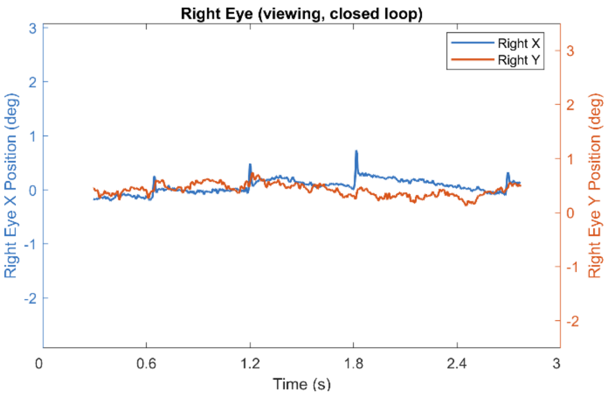

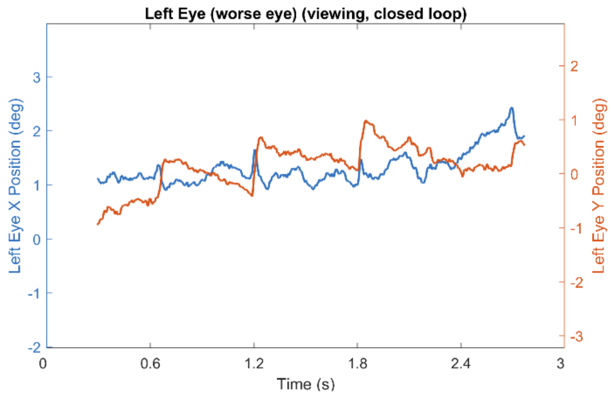

Fellow eye viewing: nystagmus

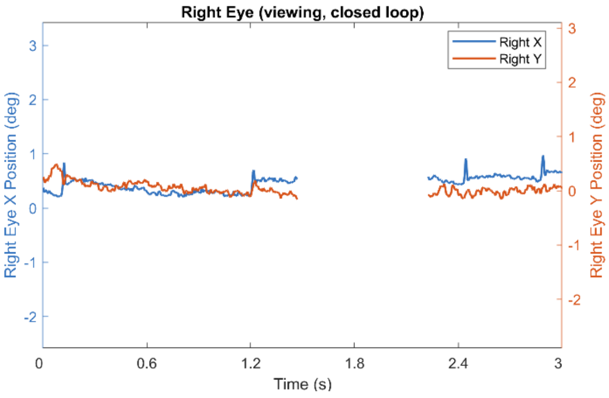

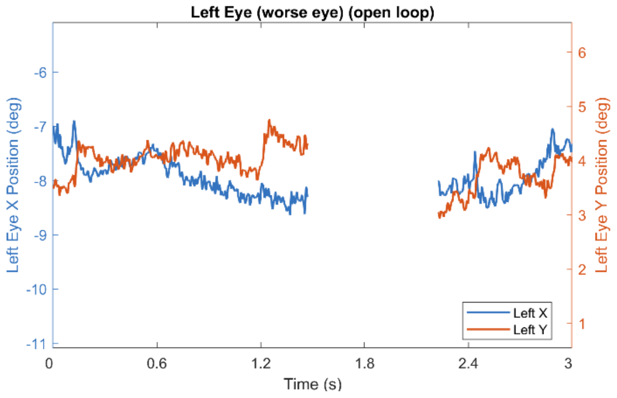

Amblyopic eye viewing: nystagmus

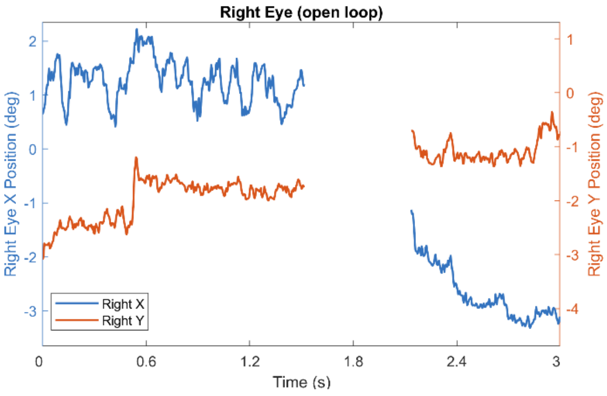

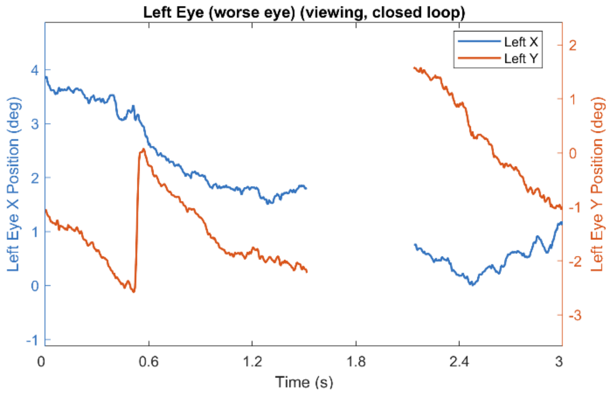

P6

Binocular viewing: nystagmus *(amblyopic eye data not available)*

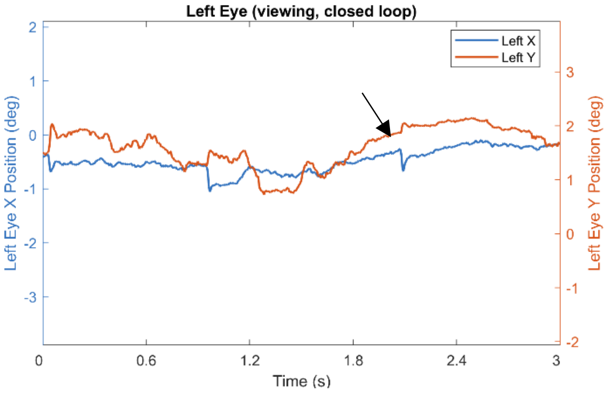

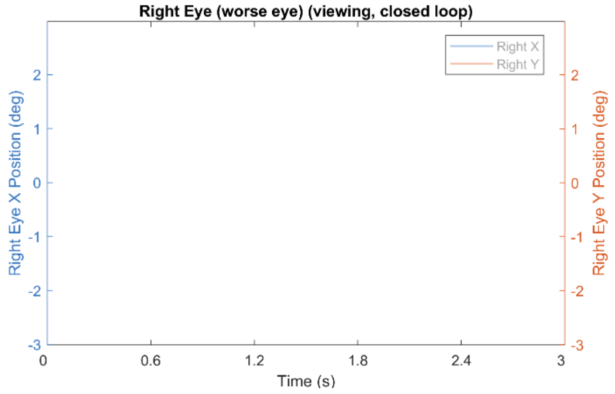

Fellow eye viewing: nystagmus *(amblyopic eye data not available)*

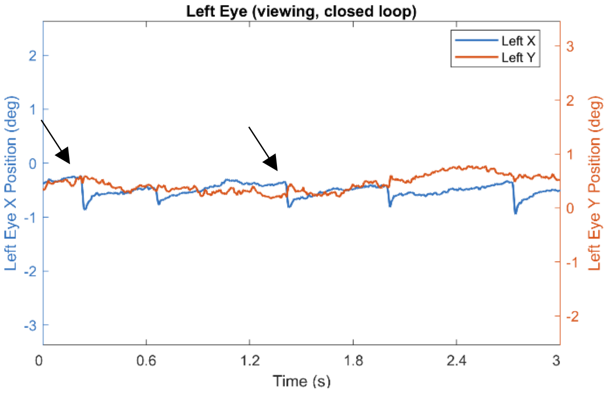

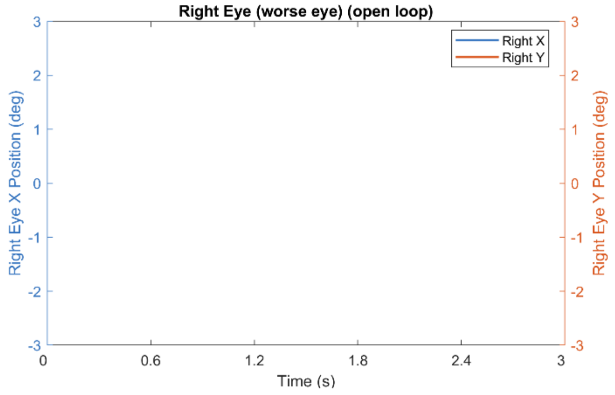

Amblyopic eye viewing: dissociated nystagmus (frequencies and quick phase directions varied between eyes)

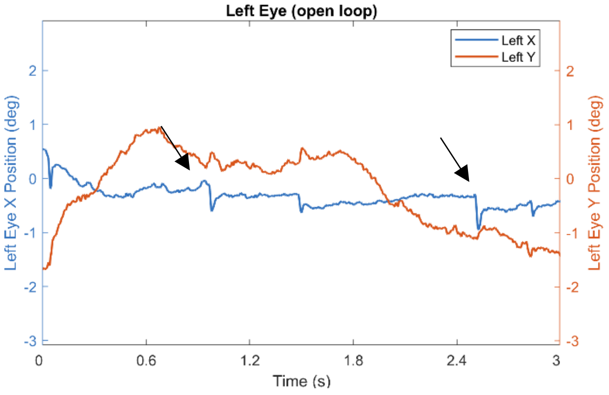

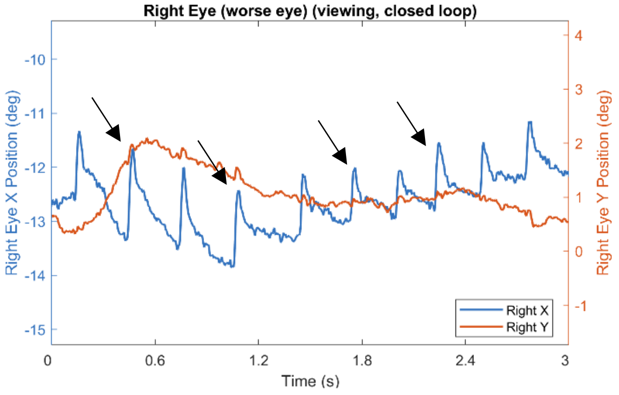

P7

Binocular viewing: nystagmus *(amblyopic eye data not available)*

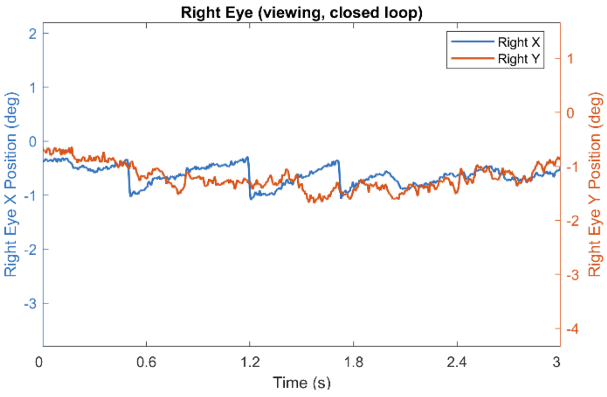

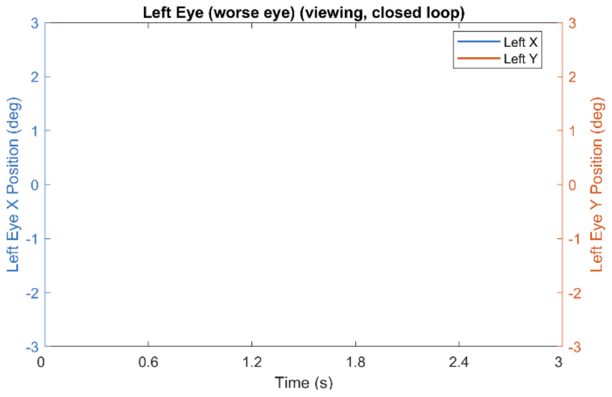

Fellow eye viewing: nystagmus *(amblyopic eye data not available)*

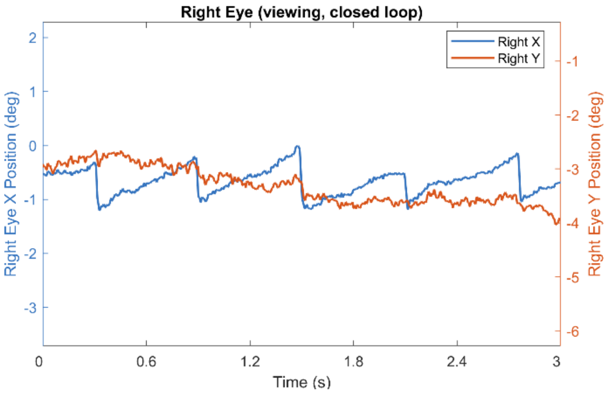

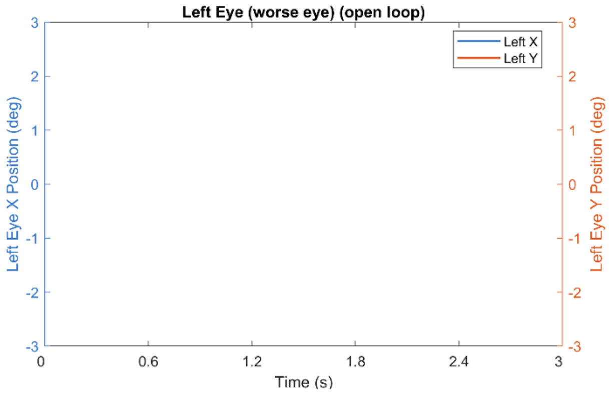

Amblyopic eye viewing: nystagmus

Note: the y range of this graph is doubled (12 degrees) due to large left eye movements.

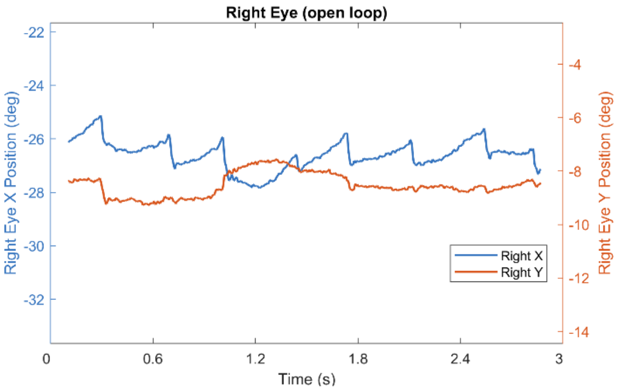

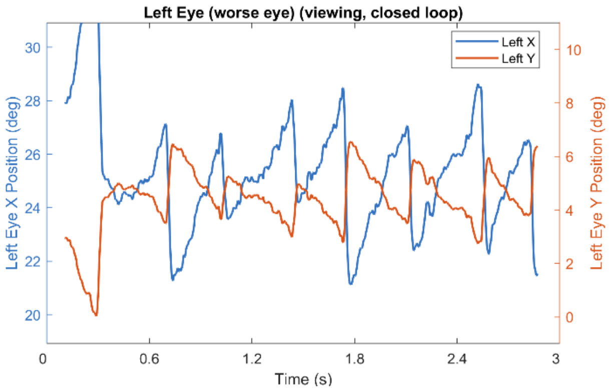

Supplement: Supplementary file 2 [file Supplementary_file_2.pdf]
